# Recommendations for Dental Care during COVID-19 Pandemic

**DOI:** 10.3390/jcm9061833

**Published:** 2020-06-12

**Authors:** Katarzyna Gurzawska-Comis, Kathrin Becker, Giulia Brunello, Agata Gurzawska, Frank Schwarz

**Affiliations:** 1 Department of Oral Surgery, University of Birmingham, 5 Mill Pool Way, Edgbaston, Birmingham B5 7EG, UK; k.a.gurzawska@bham.ac.uk; 2 Department of Orthodontics, Universitätsklinikum Düsseldorf, Moorenstr. 5, Building 18.21, 40225 Düsseldorf, Germany; 3 Department of Oral Surgery and Implantology, Carolinum, Goethe University, Theodor-Stern-Kai 7/Haus 29, 60596 Frankfurt am Main, Germany; f.schwarz@med.uni-frankfurt.de; 4 Department of Neurosciences, Dentistry Section, University of Padova, Via Giustiniani 2, 35128 Padova, Italy; giulia-bru@libero.it; 5 Trilateral Research Ireland, Marine Point, Belview Port, X91 W0XW Waterford, Ireland; agata.gurzawska@trilateralresearch.com

**Keywords:** COVID-19, SARS-CoV-2, coronavirus, patient questionnaire, patient triage, dental emergency treatment

## Abstract

Dental clinics were suspected to be a hotspot for nosocomial transmission of coronavirus disease 19 (COVID-19), yet there has been no clear recommendation about emergency dental care and appropriate personal protective equipment during pandemics. In this paper, we aim to summarize recommendations for (i) patient risk assessment, (ii) patient triage, and (iii) measures to prevent infection of health professionals and nosocomial transmission in dental clinics. The available evidence was collected by performing searches on PubMed, Embase, and Cochrane databases. We reviewed papers on COVID-19, severe acute respiratory syndrome (SARS), Middle East respiratory syndrome (MERS), influenza, and related respiratory viral diseases. Legal and ethical frameworks, as well as international (e.g., World Health Organization (WHO)) and national (e.g., public health institutes, dental associations) guidelines were screened to summarize recommendations related to dental emergency care. To assess the patient risk, a questionnaire was developed to classify patients at unknown, high, and very high risk. Patient triage recommendations were summarized in a flow chart that graded the emergency level of treatments (i.e., urgent, as soon as possible, and postpone). Measures to prevent disease transmission based on current evidence were grouped for dental health professionals, dental clinics, and patients. The present recommendations may support health professionals implement preventative measures during the pandemic.

## 1. Introduction

A novel coronavirus disease 19 (COVID-19) caused by a β-coronavirus (Severe acute respiratory syndrome coronavirus 2 (SARS-CoV-2)) was first reported in December 2019 (Wuhan, China). On 11 March 2020, COVID-19 was declared a public health emergency of international concern by the World Health Organization (WHO). As of 5 June 2020, the Johns Hopkins University website reported that there were 6,627,287 confirmed cases worldwide and 391,686 total deaths across 188 different countries. The true number of infected people and deaths, however, is estimated to be even higher [1].

Specific features of the novel coronavirus account for its high transmissibility and pathogenicity. The virus can be directly transmitted through cough, sneeze, and respiratory droplets, as well as by contact-transmission [2,3,4]. Patients are contagious prior to the onset of clinical symptoms [5] and the contagiousness can last up to three weeks after recovery [6,7]. Moreover, patients with very mild or subclinical symptoms were found infective [8]. As incubation periods can exceed 14 days [9] and only a few patients are tested due to limited capabilities, detecting contagious people is challenging. Virus aerosol viability of >3 h and high surface stability of >72 h may further account for a high risk of nosocomial transmission [10].

Recently, the highest infection potential among all health care professionals was reported for dentists, dental assistants, and dental hygienists, as they are in close contact with patients, and exposed to spatter of patient’s secretions, saliva, and aerosol [11]. Hence, dental offices may be a relevant hotspot for virus transmission, putting health professionals at high risk of SARS-CoV-2 infection and patients at risk of nosocomial infection.

Meng et al. were the first to report on personal protection equipment (PPE) that protected dental professionals during the COVID-19 epidemic in Wuhan, China [12]. At that time, all private offices were closed in the Hubei region to prevent nosocomial infection, hospitals were restricted to emergency treatment, and dentists were equipped with PPE. In other countries, such as Poland and Germany, elective treatment is not prohibited and due to a lack of clear recommendations, dentists have to evaluate whether to close their offices, how to communicate with patients, and what type of PPE to buy.

The aim of this work is to summarize recommendations for (i) individual risk assessment, (ii) patient triage, and (iii) measures to prevent health professionals from infection and nosocomial transmission at dental clinics. The study also aimed to synthesize the related legal and ethical frameworks justifying the proposed recommendations.

Present recommendations are provided to serve dentists to implement appropriate measures for minimizing the risks of nosocomial COVID-19 transmission and to prevent infection of dental health professionals.

## 2. Experimental Section

The present recommendations were prepared after reviewing the available literature and guidelines regarding COVID-19, severe acute respiratory syndrome (SARS), Middle East respiratory syndrome (MERS), pandemic influenza, and related respiratory viral diseases. As public health emergencies also pose ethical, social, and legal dilemmas, available expert statements were also assessed.

### 2.1. Composition of the Recommendation Development Group

The steering group who developed these recommendations is composed of dental researchers and clinicians with several years of expertise in systematic review and literature searches. In addition, one lawyer was included to address concerns related to dental emergency care from a legal and ethical framework.

### 2.2. The End-User of the Recommendations

The present recommendations are intended for dental health professionals, hospital managers, and politicians.

### 2.3. Data Searches

PubMed, Embase, and Cochrane databases were searched using the following terms: “COVID-19”, “coronavirus”, “dental emergencies”, “personal protective equipment”, “SARS”, “MERS”, “dentistry”, “triage”, “medical ethics”, “bioethics”, “right to health”, “dentist code of ethics”, “human rights”, and “pandemics”. A hand search was performed to consider the preventive measures specified in Section 2.4. Due to the topic’s novelty, studies in English, German, Italian, and Polish were screened by the authors. No publication type restrictions were applied. The screening for relevant literature was limited up to 1 June, 2020. Due to the recent onset of COVID-19, expert’ opinions summarized in international guidelines were also considered [13]. Furthermore, legal and ethical frameworks, as well as national (e.g., dental societies, public health institutes) and international guidelines (e.g., WHO, European Centre for Disease Prevention and Control, Infection prevention and control for COVID-19, Council of European Dentists) related to dental emergency care were screened.

### 2.4. Synthesis of Literature

The available literature was synthesized according to the following topics:

1. Virus transmission and pathogenicity.
2. Testing.
3. Assessment of individual COVID-19 risk.
4. Classification of emergency level for dental treatments.
5. Measures by health care professionals to limit virus spread, including masks, face shields, coats, gloves, and double shoes.
6. Measures to limit nosocomial infection at dental clinics, including disinfection, isolation rooms, ventilation, waiting rooms, dining, and locker rooms.
7. Measures applied by patients including overshoes, masks, and mouth rinses.
8. Information collected from legal and ethical frameworks relevant to COVID-19 pandemic in dentistry.

## 3. Results

### 3.1. Virus Transmission and Pathogenicity

The transmission mechanism of SARS-CoV-2 has not yet been fully understood. Initially, the proposed main transmission routes were direct, through cough, sneeze, and respiratory droplets, as well as contact with mucous membranes of eyes, nose, or oral cavity [12,14,15]. Recent studies demonstrated high loads of virus RNA in hospital aerosol [16]. Moreover, particles generated by asymptomatic carriers while speaking are increasingly considered to be a major mode of transmission as the virus remained suspended in air for 10s of minutes or even longer [17,18,19]. Hence, 9 cases out of 10 of SARS-CoV-2 transmission has been estimated to be airborne. This is in-line with recent findings revealing substantial virus load in the oral mucosa and in stimulated saliva from submandibular gland duct [20,21]. Comparable virus loads were reported for children and adults [22], thus suggesting that children may be as infectious as adults.

The median incubation period was reported to be 5.1 days [95% CI: 4.5–5.8 days], however the incubation periods can exceed 14 days [9]. Patients were shown to be contagious prior to the onset of clinical symptoms [23]. Moreover, patients presenting with very mild or subclinical symptoms were found infective [4,6,7] and the contagiousness could persist up to three weeks after recovery [8,24]. The ratio of asymptomatic to symptomatic patients was estimated to be 41–42% [25,26].

SARS-CoV-2 was reported to be airborne through medical aerosols [27]. In addition, virus viability in the aerosol was shown to exceed 3 h and surface stability of the virus exceeded 72 h [10].

### 3.2. Testing

At present, COVID-19 testing can identify the SARS-CoV-2 virus and two methods are most frequently applied, i.e., nucleoid acid and antibody detection techniques. The first method using real time polymerase chain reaction (RT-PCR) indicates the presence of the virus in the tested sample. The second one detects antibodies produced in response to infection. Another option to identify the COVID-19 is assessment of lung damage via either a computed tomography (CT) scan or low oxygen uptake saturation. Antigen detection [18] and isothermal amplification assays [28,29,30] are considered the fastest methods for detection of COVID-19. However cross-reactivity with other seasonal flu coronaviruses has been reported, which decreases specificity of a positive outcome [31,32,33].

The RT-PCR is currently considered as the “gold standard” and is characterized by rapid detection, high sensitivity, and specificity [34]. The sample for RT-PCR is obtained by a nasopharyngeal swab or sputum, as well as saliva [35]. Within a recent study by Wyllie et al., 2020, saliva and nasopharyngeal swab samples collected from inpatients and co-workers were compared [35]. The results indicated that saliva yielded greater detection sensitivity and consistency throughout the course of infection. Furthermore, the authors suggested that saliva may be more sensitive for detecting asymptomatic or pre-symptomatic infections. Further studies confirmed higher sensitivity of saliva as a diagnostic specimen compared to nasopharyngeal swabs [36,37,38,39].

### 3.3. Individual Risk Assessment

Clinical manifestations including flu-like symptoms, dry cough, fever, shortness of breath, anosmia, or headache are frequently associated with COVID-19. In addition, patients who recently travelled to COVID-19 high risk countries (WHO) and/or having contact with COVID-19 positive patients during the last 14 days exhibited an elevated risk of infection. Furthermore, asymptomatic patients without travel history/contact with COVID-19 positive patients may be contagious and their risk may be considered unknown [9]. The epidemiological history [40] and clinical manifestation [41] assessment was therefore suggested to identify patients with suspected risk of COVID-19. The Polish Dental Association (PTS) [42] and American Association of Endodontists proposed questionnaires to classify individual patient risk [43]. The risk of severe disease associated with COVID-19 for people in the EU/EEA, Switzerland, and UK is currently considered moderate for the general population and very high for older adults and individuals with chronic underlying conditions [44,45]. As the clinical situation may vary over time, a screening process over the phone has been proposed to be confirmed at the dental office [46,47,48].

### 3.4. Classification of Emergency Level for Dental Treatments

The various national dental guidelines published by the American Dental Association, the Polish Dental Association, the Società Italiana di Parodontologia e Implantologia, and the Swiss Association of Dentists, recommend avoiding elective treatments during the COVID-19 pandemic [42,45,49,50]. A triage is considered fundamental to prevent nosocomial transmission and infection of health care professionals. Few guidelines further specify the emergency level for dental treatments [45,49,51]. The Scottish recommendation proposed emergency, urgent, and routine care [51]. At this point it has to be emphasized that emergency usually implies life-threatening conditions, which are not-commonly performed by dentists but by oral and maxillofacial surgeons within hospital settings. Most guidelines recommended assessing dental treatment needs using telephone and video review as the primary route.

### 3.5. Measures by Health Care Professionals to Limit Virus Spread, Including Masks, Face Shields, Coats, Gloves, Double Shoes

#### 3.5.1. Use of Masks

Due to the direct transmission of SARS-COV-2, masks were found to be an effective measure to prevent infection (difference mask versus no mask: 4.65%, *p* < 2.2 × 10^−16^) [52].

The efficacy of N95 respirators against surgical masks was examined in a systematic review and meta-analysis [53]. After performing a sensitivity analysis, the authors identified a beneficial effect of N95 respirators on preventing respiratory viral infection compared to surgical masks (relative risk (RR) = 0.61, 95% CI 0.39–0.98, *p* < 0.05). Furthermore, N95 respirators significantly reduced bacterial colonization in hospitals (RR = 0.58, 95% CI 0.43–0.78, *p* < 0.05). One in vitro study demonstrated that a tightly sealed surgical mask blocked 94.5% of total virus whereas a tightly sealed respirator blocked 99.8%. In contrast, a poorly fitted respirator blocked only 64.5%. A simulation of how masks are worn by healthcare professionals revealed that 68.5% of total viruses were blocked. These results indicate that a poorly fitted respirator performs no better than a loosely fitting surgical mask [54].

The US National Institute for Occupational Safety and Health (NIOSH) classifies particulate filtering facepiece respirators (FFRs) depending on their capacity to filter oil droplets into nine categories: N, not resistant to oil (N95, N99, N100); R, somewhat resistant to oil (R95, R99, R100); and P, strongly resistant to oil (P95, P99, P100). The numerical identifier corresponds to the minimum filtration efficiency (i.e. 95%, 99%, and 99.97%). The European Standard (EN 149:2001) defines three classes of filtering face pieces (FFP), with minimum filtration efficiencies of 80% (FFP1), 94% (FFP2), and 99% (FFP3). Hence, FFP2 are considered equivalent to N95 and FFP3 to N99 [55].

The WHO recommended in its interim guideline on COVID-19 the use of NIOSH-certified N95, European Union (EU) FFP2, or equivalent masks, when aerosol-generating procedures are performed [56]. In the case of short storage PPE, the cause of N95 respirator was proposed to be reused, with multiple removals between patients [57].

Concerns have been recently raised about use of FFP3 masks with valves, due to the increased risk of COVID-19 transmission for the patient through unfiltered exhaled clinician’s breath. Therefore, it has been suggested to combine the use of surgical masks covering FFP3 with a valve [58]. This should be applied also to FFP2 masks with valves.

#### 3.5.2. Use of Face Shields/Goggles

Face shields and goggles are commonly considered as adjunctive measures combined with either surgical masks or N95/FFP2/FFP3 respirators.

An in vitro study [59] reported, face shields provide about 96% prevention immediately after exposure to influenza-laden cough aerosol. This value decreased to 23% when the aerosol had dispersed throughout the room.

Therefore, goggles may provide increased eye protection [60].

#### 3.5.3. Additional Measures (Gowns, Overshoes, Gloves)

Additional PPE including gowns, overshoes, and double-gloves have been suggested for use [12]. However, there is low-certainty evidence that double gloving might decrease the risk of contamination [61]. Hand disinfection agents of limited antiviral activity are effective [62].

### 3.6. Measures to Limit Nosocomial Infection at Dental Clinics, including Disinfection, Isolation Rooms, Ventilation, Waiting Rooms, Dining, and Locker Rooms

#### 3.6.1. Disinfectants against Human Coronavirus

SARS-CoV-2 was found to maintain stability on copper surfaces, ceramics and glass up to 4–5 h, on gloves up to 8 h, and on carton plastic and steel from 24 h up to 3 days [10]. Disinfectants containing ethanol (78%–95%), iodopovidone solution (0.23%–7.5%) inactivated high-concentration coronavirus within 30 s to 1 min [5,6,8]. To effectively inactivate SARS-CoV-2 virus with sodium hypochlorite, a minimum concentration of 0.21% (applied for 30 s) was required [63].

#### 3.6.2. Ventilation

Very few studies have evaluated the association of ventilation on disease transmissibility. A positive effect of (natural) ventilation has been suggested [64], which reduced pathogen transmission [65]. The WHO guideline 2009 on natural ventilation for infection control in healthcare settings recommended an hourly average ventilation rate of 160 l/s/patient for airborne precaution rooms [66].

#### 3.6.3. Isolation Rooms

Isolation rooms are commonly used to exclude patients with highly infectious diseases. Meng, Hua, and Bian [12] suggested negative pressure isolation rooms for dental emergency care for COVID-19+ patients and aerosol-generating procedures. No further evidence was found.

#### 3.6.4. Air Disinfection

Ultraviolet (UV) air disinfection is commonly used to control airborne virus transmission [67]. UVC disinfection of viral aerosols effectively inactivated coronavirus [68], which was also confirmed for SARS coronavirus strain CoV-P9 in culture medium [69]. UVA was not effective [70].

### 3.7. Measures Applied by Patients including Hand Hygiene, Overshoes, Masks, and Mouth Rinse

#### 3.7.1. Hand Hygiene

Handwashing with soap was found to be the most effective for elimination of enveloped viruses (such as SARS-CoV-2), especially when compared with alcohol-based hand rubs [71].

#### 3.7.2. Masks

A tendency towards increased use of masks has been observed in countries affected by the COVID-19 epidemic [72]. Surgical mask worn by infected patients was shown to limit virus transmission [73,74]. Since asymptomatic COVID-19 patients can be highly contagious, use of masks may be an adjunctive measure to limit virus transmission [75].

#### 3.7.3. Other Measures

Additional adjunctive measures including locking personal belongings and wearing disposable overshoes have been recommended [47,48,50], since sources of bioaerosols can arise from human clothes or furniture [76]. Due to the risk of airborne transmission, eliminating the number of patients inside the dental clinic or keeping a distance of at least 1 m in waiting areas has also been advised [55].

#### 3.7.4. Mouth Rinse

Preoperative mouthwash containing oxidative agents, such as 1–1.5% hydrogen peroxide or 0.2% povidione iodine, were reported to be viricide [77,78]. Antiseptic mouth rinses, e.g., Chlorhexidine (0.2%) and Listerine^®^ (with adjunctive alcohol) may also exhibit viricide properties [79,80]. In contrast, Chorhexidine (0.1% and 0.2%) without adjunctive alcohol are suspected not to be effective against viruses [78,81]. Cetylpyridinium chloride (CPC) may possess viricide activity especially against enveloped viruses [82,83]. Flavonoids and β-Cyclodextrin-Citrox compounds were also suggested to be effective [81,84]. However, there is a lack of evidence to what extent adjunctive compounds could elevate the substantivity of the rinses.

As the virus reproduces in the salivary gland ducts (which express the ACE-2 receptor) [85], the virus load of saliva was reported to be high [86]. Since saliva is constantly produced, it is not yet clear how long the antiviral effect of mouthwashes may last.

### 3.8. Aspects Collected from Legal and Ethical Frameworks Relevant to COVID-19 Pandemic in Dentistry

The COVID-19 pandemic raises ethical and legal questions including priorities in the allocation of resources and access to medical care. The four principles of medical ethics namely respect for autonomy, beneficence, nonmaleficence, and justice, may support dentists in making decisions when reflecting on moral issues that arise at work during pandemics.

Dentists have a duty to act in the best interests of patients and prioritize their interest (principle of beneficence) while respecting their dignity, autonomy, and choices (principle of autonomy) [87]. COVID-19 threatens particularly vulnerable groups such as the elderly, individuals with underlying systemic diseases (e.g., cardiovascular disease, diabetes, chronic respiratory disease, and hypertension). or individuals living in socially deprived circumstances. During pandemics, personal human dignity remains the highest of all values, it is independent from social standing or of usefulness to others [88]. Hence, treatments should be provided regardless a patient’s age, sex, national or ethnic origin, color, place of residence, social, or economic status (principle of justice). Accordingly, patients should be admitted for the urgent treatment based on strictly medical prognosis. The COVID-19 amplifies the problems to access to medical care and issues related to scarce resources. Therefore, it is ethically justified to re-schedule or cancel elective procedures. Nevertheless, dentists should remain available to manage urgent treatment needs [49,55,89].

Dentists have a duty to provide the most essential and appropriate care and to treat patients according to their needs [87]. Nevertheless, during pandemics the duty to treat or to care may need to be balanced with doctors’ right to life and to protect their own, their family, and dental team’s life and health [90,91].

## 4. Recommendations

Based on currently available evidence and international guidelines, the following recommendations are proposed.

### 4.1. Individual Risk Assessment

We recommend that patients who had contact with COVID-19 positive patients or visited any high-risk region according to the WHO in the last 14 days should be classified as very high-risk patients.

We recommend that patients who had contact with COVID-19 positive patients or visited any high-risk region according to the WHO in the last 14 days, but do not show flu-like symptoms should be classified as very high-risk patients.

We recommend that patients who did not have contact with COVID-19+ patients or did not visit any high-risk region according to the WHO in the last 14 days but show flu-like symptoms should be classified as high-risk patients.

All other patients should be classified as having an unknown risk, i.e., being potentially contagious.

A questionnaire to evaluate risk assessment is provided in File S1. The outcomes of the questionnaire are recommended to be translated into the individual risks as follows:

– Very high risk: The patient scores at least 1 YES in epidemiological history AND/OR patient has been tested positive for COVID-19
– High risk: The patient scores at least 0 YES in epidemiological history and at least 1 in clinical manifestation
– Unknown risk: The patient scores 0 YES in epidemiological history and 0 YES in clinical manifestation.

#### Rationale of the Authors

Due to the high number of asymptomatic patients and the contagiousness prior to and after the onset of clinical symptoms, the authors recommend that every patient has to be considered potentially contagious during the COVID-19 pandemic. However, particular precaution is advisable for patients with a very high likelihood of contagiousness.

### 4.2. Patient Triage Recommendation

**Figure 1:**
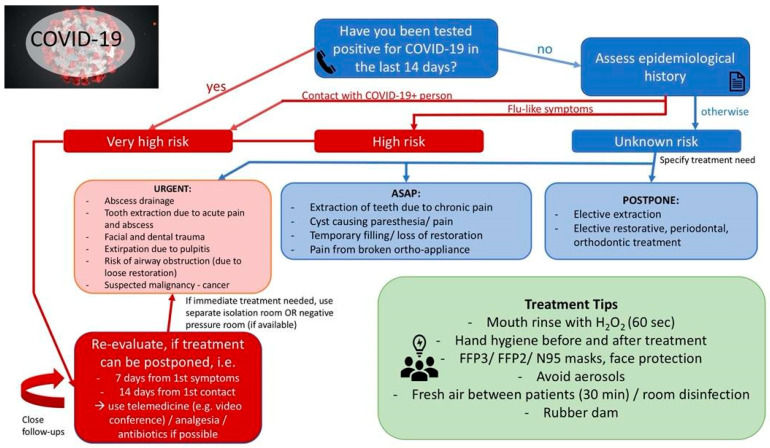
Patient triage recommendations (diagram). COVID-19, coronavirus disease 19; ASAP, as soon as possible.

We recommend classifying dental treatment needs as urgent (within the next 24 h), as soon as possible (ASAP) (within the next 7 days) and postpone (no urgency). The recommendations are summarized in Figure 1. For the patient triage, we divided the recommendations into general, as well as COVID-19 risk specific (see Section 4.1).

#### 4.2.1. General Recommendations

Prior to the visit, we recommend assessing individual patient risk and dental treatment needs at the telephone or through a video conference.

To limit the number of potentially contagious people in the clinic, the patient should not report with accompanying people. Exceptions comprise disabled, children, or geriatric and hospitalized patients. Furthermore, aerosol free treatments should be selected (if possible). When radiographs are needed, extraoral radiographs should be preferred. Rubber dam may be used when applicable to limit virus transmission.

#### 4.2.2. Triage Recommendations

Patients exhibiting very high risk should generally not be seen at the clinic unless they have an urgent treatment need (video conference may be used to validate urgent treatment needs). Otherwise, antiseptics or antibiotics in combination with frequent telephone interviews may be used to postpone the treatment until recovery.

Questions to identify urgent treatment needs may include:

1. Do you have pain? Yes/No
2. If yes, where is the pain and from how long? Yes/No
3. Is the pain associated with swelling and limited opening of the mouth? Yes/No
4. Have you taken any medication, like paracetamol/ibuprofen/Aspirin? If yes, did you find any relief? Yes/No
5. Do you have any underlying medical conditions? If yes, which one.

##### Rationale of the Authors

Based on legal and ethical frameworks, dental treatments have to be provided to all patients in demand or pain. During the COVID-19 pandemic, social distancing is mandatory to minimize the spread of the virus and there is a risk of nosocomial transmission at dental clinics. Therefore, we recommend avoiding dental treatments unless they cannot be postponed. However, concerns may arise regarding the unnecessary overprescribing of antibiotics from tele-dentistry, thus contributing to the development of antibiotic resistance.

### 4.3. Measures to Prevent Infection in Dental Settings

**Figure 2:**
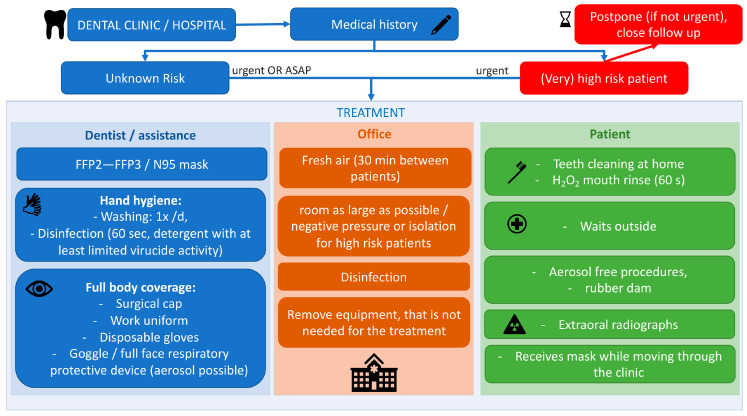
Measures to prevent health professional infection and nosocomial transmission at dental clinics (diagram). FFP, Filtering Face Piece.

We recommend the following measures to be adopted during COVID-19 pandemic. The measures are summarized in Figure 2.

#### 4.3.1. Personal Protective Equipment for Dental Professionals

For patients exhibiting a (very) high COVID-19 risk, we recommend using N95/FFP2 or FFP3 masks with proper fit, face-shields, and regular gloves. Adjunctive use of caps, overshoes, and coats may further increase safety. The same measures are recommended to be used for patients exhibiting an unknown risk, whereas a regular surgical mask may be combined with a face-shield for aerosol-free treatments in scarcity of PPE.

#### 4.3.2. Recommendations Regarding the Dental Clinic

Between consecutive patients, we recommend proper disinfection and sufficient room ventilation (time depends on the size of the room, the number and the size of the windows, the type of treatment). For room disinfection, ethyl-alcohol (78%) is considered to be the most effective. Disinfection should include all flat surfaces, computers, keyboard, mouse, lamps, and doorknobs.

Negative pressure isolation rooms may be considered to treat patients with COVID-19 or very high risk of disease to prevent virus-laden aerosols transmission. However, it has to be noted that isolation rooms are only effective when the airflow direction is well controlled by adequate pressure monitors and negative pressure control devices.

#### 4.3.3. Recommendations to Be Followed by Patients

We recommend patients attending dental clinics must perform hand hygiene using alcohol-based hand rub or soap and water. Patients should store their belongings in a locker outside the clinic and wear overshoes (if available). They should be instructed to refrain from touching their mouth, nose, and eyes with dirty hands, and they should avoid shaking hands. If outdoor waiting is inevitable, patients are recommended to maintain at least a 1 m distance in the waiting areas. Prior to the treatment, we recommend rinsing with a mouth wash to reduce the virus load in saliva prior to beginning dental treatment. Hydrogen peroxide (1–1.5%) or povidione iodine (0.2%) were reported to be most effective.

##### Rationale of the Authors

Due to the high risk of nosocomial transmission and as every person is potentially contagious, maximum prevention measures are required to prevent direct-contact transmission, but also indirect transmission of the virus.

### 4.4. Future Perspectives for Elective Dental Care

We recommend that patients, before visiting the dental office, have epidemiological and clinical screening. Ideally, patients and health professionals should also be tested for COVID-19 before dental treatment. Unless COVID-19 test of a patient has been negative, elective treatments should be performed following the infection control measures presented in above recommendations. Briefly, if the treatment includes aerosol generating procedures, the FFP2/FFP3 masks and other PPE should be used. If the treatment does not include aerosol, surgical mask with visor may be used unless there are other risks such as underlying medical conditions.

## 5. Conclusions

The risk of COVID-19 infection by dental staff and also the risk of nosocomial transmission in dental practices is considered high. The reasons are the short distance to the patient, proximity to saliva, blood, spatter and aerosol exposure. In many countries affected by COVID-19, elective dental treatments were suggested to be postponed. Since every patient can be potentially contagious, increased safety measures were suggested to all patients when treatment is mandatory.

As neither dentists nor the (inter-)national hygiene guidelines appeared to be prepared for the present pandemic and to limit infection and COVID-19 transmission in dental clinics, the present article proposed recommendation on the following topics: (i) individual risk assessment, (ii) patient triage, (iii) measures to prevent infection of health professionals’, and nosocomial transmission at dental clinics.

However, limited evidence was available regarding COVID-19. Therefore, the present recommendations were also based on evidence from similar diseases, such as SARS and MERS.

The described recommendations were suggested for the COVID-19 pandemic only. Before vaccination is available, less strict recommendations may be required to prevent infection and nosocomial transmission in dentistry.

Furthermore, rapid test kits are becoming available and their extensive use prior to dental care provision might help in determining the health status of the patients. As the duration of the pandemic cannot be predicted at the moment and elective treatments cannot be indefinitely postponed, testing may represent a positive turning point in dental patients’ management during pandemic, thus allowing to safely provide elective treatments, also involving aerosol-generating procedures, in COVID-19 negative patients.

## Supplementary Materials

The following are available online at https://www.mdpi.com/2077-0383/9/6/1833/s1, File S1: Questionnaire for risk assessment.
