# Supplementary material for: Recommendations for Dental Care during COVID-19 Pandemic
Source: J Clin Med. 2020 Jun 12;9(6):1833. doi: 10.3390/jcm9061833 (PMC7357003; doi:10.3390/jcm9061833)
Supplement: Supplementary file 1 [file jcm-09-01833-s001.pdf]

File S1: Questionnaire for risk assessment.

# Epidemiological screening COVID-19

**Patient/carer details**
**NAME:**
**SURNAME:**
**DATE OF BIRTH:**
**Personal/Hospital number:**

**Declaration:**

Please declare the following:

| | | | |
|---|---|---|---|
| 1 | Within 14 days, have you traveled or residence in the high-risk regions or countries listed by WHO? | YES | NO |
| 2 | Within 14 days, have you had contact with those infected with coronavirus (COVID-19 positive)? | YES | NO |
| 3 | Within 14 days, have you had direct contact with those with fever or respiratory symptoms? | YES | NO |
| 4 | Within 14 days, have you had contact with those who were under quarantine? | YES | NO |
| 5 | Have you had symptoms of COVID-19 (fever, cough, short breaths, other flu-like symptoms)? | YES | NO |
| 6 | Has anyone from your family members or housemates or work colleagues had symptoms as above? | YES | NO |
| 7 | Have you taken paracetamol during the last 12 hours? | YES | NO |
| 8 | Have you taken ibuprofen during the last 12 hours? | YES | NO |
| 9 | Have you taken aspirin during the last 12 hours? | YES | NO |
| 10 | Have you taken any other medications for pain relief? If yes, please list: | YES | NO |

In the European context, this questionnaire should be conducted in accordance with the General Data Protection Regulation (GDPR) (EU) 2016/679 and the national data protection regulation.

**City………………...** **Date………………...** **Signature………………...**
